# Phonological remediation effects on a child with giftedness and developmental dyslexia

**DOI:** 10.1590/2317-1782/20242023068en

**Published:** 2024-08-05

**Authors:** Alba Miranda Beserra Gurgel Sena, Bárbara Louise Costa Messias, Roberta Louise Mariano Bezerra, Anna Irenne de Lima Azevedo, Hellen França Alcantara, Cíntia Alves Salgado Azoni

**Affiliations:** 1 Programa Associado de Pós-graduação em Fonoaudiologia – PPGFon, Federal University of Rio Grande do Norte – UFRN - Natal (RN), Brasil.; 2 Graduate Program of Psychology, Federal University of Rio Grande do Norte – UFRN - Natal (RN), Brasil.; 3 Departament of Speech, Language and Hearing Sciences, Federal University of Rio Grande do Norte – UFRN - Natal (RN), Brasil.

**Keywords:** Language Therapy, Case Reports, Reading, Reading Development Difficulty, Handwriting

## Abstract

Twice-exceptionality is characterized as the presence of high performance concomitantly with deficiencies or incompatible conditions. An example is when giftedness manifest associated with neurodevelopmental disorders. This study is a clinical case report referring to the evaluative and interventional process of a 9- year-old child with the paradoxical combination of giftedness associated with dyslexia. It aims to compare the performance in phonological processing, reading and writing before and after phonological remediation. In the first assessment, the child demonstrated alphabetic level in reading, a transition phase between syllabic-alphabetic and alphabetical writing levels, and below-expected performance in phonological processing skills. After intervention, the results showed consistent improvements in phonological processing, the consolidation of alphabetical writing and orthographic reading level. In general, children with isolated dyslexia have persistent difficulties in several skills after intervention. The evolution shown after phonological remediation, especially at reading level, shows different characteristics than expected. Thus, it can be concluded that twice-exceptionality may have favored the overcoming of some of the shown difficulties more successfully. Studies on these combined conditions can contribute to a better understanding of this framework during the development of learning and to formulate specialized interventions.

## INTRODUCTION

Twice-exceptionality (2E) can be characterized by a high performance in one or more areas, concomitant with a psychiatric, educational, sensory or physical disorder. It is classified as a mediated development between the individual's coexisting potential and weakness, generating qualitatively diverse outcomes^(1)^. As such, children may simultaneously present high abilities/giftedness (HA/G) and neurodevelopmental disorders, such as: Attention-deficit/hyperactivity disorder (ADHD), autism spectrum disorder (ASD), learning disabilities (LD), among others^(2)^.

This past decade has been marked by the difficulty of recognizing twice exceptional children and adolescents, especially due to society's myths and ill-conceived notions regarding high abilities/giftedness (HA/G)^(3)^.

Historically, HA/G may convey different theoretical models used to further comprehend it. The Renzulli three-ring conception of giftedness is one of the most significant contributions for HA/G literature and will be used herein to better understand HA/G. In this theoretical conception, HA/G are identified from 3 perspectives: creativity, intelligence (above average capacity) and task commitment^(4,5)^.

In the present case report, the paradoxical combination between HA/G and a specific learning disability with reading impairment, dyslexia, will be evidenced.

Dyslexia is characterized within the group of Specific Learning Disabilities. In the case of specific learning disability with reading impairment, or dyslexia, the child may present persistent problems with word reading accuracy, reading fluency and comprehension, reading speed, writing and spelling difficulties^(6)^. Consequently, this condition is associated with academic skills that are far below average for age and educational level^(7)^.

Therefore, in order to understand more accurately the child's performance and reading skills difficulties, it is possible to outline the student's level of reading based on Frith's theory, respectively divided into the following levels: logographic, alphabetic and orthographic^(8)^.

Twice exceptional children may present a qualitatively different academic, cognitive and social performance when compared to children whose clinical picture indicates only HA/G or with dyslexia separately. The performance is diverse and varies for each clinical case, nevertheless, when these children are evaluated, the literature indicates overall specific talents such as: high level of creativity, unusual imagination and higher order thinking skills. However, coexisting reading and writing deficits may be observed^(2,9)^.

Research has suggested that HA/G can frequently mask a poor performance in academic skills of 2E individuals with dyslexia. In other words, the potentialities may help to overcome the presented difficulties^(2,10)^. Furthermore, seeing that this is a rather incipient area, still lacking evaluation and intervention standardization, these factors may contribute to the challenge for identifying and elaborating specialized strategies, seeing that this condition is characterized by a strong heterogeneity of performance. It is essential to understand which strategies can be most effective as an interventional treatment regarding the competencies of these children, so that they can overcome their greatest weaknesses and recognize possible strengths.

Thus, the objective of the present study is to compare the performance of a child with high abilities/giftedness (HA/G) associated with dyslexia in phonological processing, reading and writing tasks before and after an intervention based on a phonological remediation program. As a result, it will be possible to generate new reflections on the theme and assist in the identification of the potential challenges that twice exceptional individuals may face.

## CLINICAL CASE REPORT

This is a case study, approved by the Research Ethics Committee (REC) under No. 1,012,635. The prior authorization of both the family members and the participants was requested upon the signing of the Informed Consent Form (ICF) and the Informed Assent Form (IAF).

The participant in the present study is a female student in the 3rd year of elementary school. During the first assessment, in 2018, the child was 8 years and 2 months old, while in the second assessment, in 2019, she was 9 years and 6 months old. The interval between the evaluations occurred due to the fact that the present study used a public service, so the laboratory was in a leave from the activities during the holidays. It is further important to consider that the appointments were held only once a week and that, during this period, there was a lack of attendance from the participant, which also extended the process. As for her clinical history, the participant was born at term and presented adequate neuropsychomotor and linguistic development. The child was born and lived in a French-speaking country until the age of 2, but was exposed to another language at home, since her parents are Brazilian Portuguese speakers. However, her first words were in the French language. Upon returning to Brazil, the participant went through two private schools. In the first school, she could not communicate, as she expressed herself only in French. After this experience, she started studying at a French school at the age of 3, still in Brazil. Over the years, the participant presented reading and writing difficulties; for this reason, she was held back the 1st year of Elementary School, at her mother's request. At the age of 6, she started studying in a Portuguese-English bilingual school. At the age of 8, she was evaluated by an interdisciplinary team of speech–language pathology and neuropsychology, verifying the diagnosis of developmental dyslexia (DD) and high abilities/giftedness (HA/G). Soon after, she was referred for a reading and writing assessment in the laboratory of choice.

Phases of the study: 4 assessment appointments for each stage - pre- and post-intervention (T1 and T2, respectively)- and 20 appointments for the phonological remediation, once a week for 60 minutes. The intervention was conducted in the second half of 2018, when the participant's parents were less engaged due to their work demands.

One-hour evaluations were performed individually. During which, tasks were carried out to assess performance in phonological processing - phonological working memory, phonological awareness and lexical access -, reading and writing.

For the child's evaluation, the following protocols were used:

- **Phonological Awareness:** to assess this ability, was applied the Phonological Awareness Sequential Assessment Instrument (CONFIAS - *Consciência Fonológica: Instrumento de Avaliação Sequencial*)^(11)^. This protocol proposes tasks related to synthesis, segmentation, rhyme, alliteration, initial and final syllable identification, exclusion and transposition. Syllabic awareness is analyzed first, consisting of nine items, then phonemic awareness is analyzed, consisting of seven items. Each correct answer is equivalent to one point, 40 being the total for syllabic awareness and 30 for phonemic awareness, with the total score of 70 points. The results should be compared with the writing hypotheses to be expected according to Blacksmith and Teberosky^(12)^. With that in mind, the following normality values were used: regarding the syllabic-alphabetic writing hypothesis, 27, 12 and 39 for the syllabic, phonemic level and total score, respectively; regarding the alphabetic writing hypothesis, 31, 15 and 46.
- **Phonological working memory:** the Phonological Working Memory Test was applied^(13)^. When applying this protocol, the evaluator must start with the nonword repetition, which is composed of 40 pseudowords. The examiner must speak each word of the list, asking the child to repeat them immediately. The participant will have two attempts to properly repeat the nonwords. Each correct answer on the first attempt is equivalent to two points, one point if the child gets it right on the second attempt and, finally, zero points if the participant doesn't respond correctly in any of the attempts. Subsequently, the evaluator must proceed to the direct and inverse order assessment of the digits test, which has an equivalent score to the pseudowords. According to the age of the participant at the time of the evaluations, the normal values of 69, 13 and 6 were used for the pseudowords, direct and inverse digits, respectively.
- **Lexical access:** the Rapid Automatized Naming (RAN) test^(14)^ was applied in the assessment and the Automatic Naming Test (TENA - *Teste de Nomeação Automática*)^(15)^ in the reassessment. Both tests aim to estimate the individual's ability to name a sequence of stimuli, in other words, it measures the child's speed to promptly verbalize a visual stimulus. Two protocols were applied, since TENA had not been published yet at the time of the first assessment. Furthermore, TENA is a cutting-edge protocol much more complete for verifying normality, as it allows to perform an analysis according to the participant's age and months. Both tests have a similar application and are divided into four boards, from which the child must name colors, objects, letters and digits. The naming process must follow the same reading directionality- from left to right and top to bottom. For T1, which used the RAN test, the normality values correspond to children aged between 8 years to 8 years and 11 months, due to the participant's age in this period, thus, it must present scores of 28, 29, 52 and 46 seconds for the subtests of digits, letters, objects and colors, respectively. For T2, the protocol's (TENA) normality values for the age of 9 years and 6 months were used, with an expected score of 35, 32, 50, 53 seconds for the subtests of digits, letters, objects and colors, respectively.
- **Reading:** the Reading Comprehension Assessment protocol for Isolated Words/Pseudowords (LPI - *Leitura de Palavras/pseudopalavras Isoladas*)^(16)^ was applied first, in which the child is asked to read aloud isolated words and pseudowords, which will be tallied into a score. 19 regular words, 20 irregular words and 20 pseudowords are typed in black arial, size 24 on a white background. The child is able to obtain a total score of 59 points, since each correct reading is equivalent to one point. After that, the Assessment Protocol for Reading Comprehension of Expository Texts was applied^(17)^. This instrument aims to evaluate reading comprehension through directed questions concerning texts that are compatible with the participant's school year. In it, the silent and oral reading patterns are evaluated and timed. With this, it is possible to verify the participant's current level of reading. Additionally, the mean number of words read per minute is calculated, allowing the verification and comparison of reading speed.
- **Writing:** to assess writing, the child was asked to elaborate a text with a theme of her interest. After finishing the story, the evaluator asked the participant to read aloud what was written. Then, the child was asked to write the LPI target words in a single sheet^(16)^, to subsequently perform a dictation of the words and pseudowords. Thereafter, a writing assessment was carried out in a qualitative way, according to the orthographic analysis of Zorzi and Ciasca^(18)^.

The remediation was based on a program used for children with dyslexia^(19)^ and included activities aimed at improving phonological skills, such as: identification of graphemes and phonemes, pairs of phonemes, pairs of syllables, word pairs, addition and subtraction of phonemes, syllabic and phonemic manipulation, rhyme, alliteration, lexical access, visual working memory, auditory working memory and reading training. In every appointment, these activities were explored in a playful way, targeting mainly the metalinguistic aspects of phonological awareness. In reading training, the participant was exposed to children's books from the Mico Maneco collection. This collection has several stories that gradually increase the words' complexity level, making it possible to follow the child's progress. The activities performed and the participant's evolution report were described in their medical record at the end of each appointment.

Upon analyzing the phonological awareness performance results, the child presented in both assessments a consistent performance regarding the writing hypotheses for each period. In the first evaluation the participant received a hypothesis of syllabic-alphabetic writing and, in the second, alphabetic, demonstrating progress. The performance score improved in both skill categories, syllabic (T1= 35; T2 = 37) and phonemic (T1= 14; T2= 20) (Table 1). The improvement seen in the 4 correct answers at the phonemic level stands out, which can be explained due to the implementation of the phonological remediation focusing on the phonemic level.

**Table 1:** Performance in phonological awareness, working memory and lexical access

Skill	Variable	T1	ET1	T2	ET2
**Phonological awareness**	Syllabic awareness	35	27	37	31
	Phonemic awareness	14	12	20	15
	Total Score	49	39	57	46
**Working memory**	Pseudowords	66	69	69	69
	Direct order digits	20	13	24	13
	Reverse order digits	4	6	12	6
Lexical access*	Digits	37	28	41	35
	Letters	37	29	29	32
	Objects	62	52	59	50
	Colors	60	46	56	53

Caption: T1 = performance in the assessment; ET1 = expected performance in the assessment; T2 = performance in the reassessment; ET2 = expected performance in the reassessment; *time in seconds

The phonological working memory results prior to the phonological remediation indicate that the child was performing below expectations for the pseudowords category, presenting 66 points in T1, with expected performance for T1 (ET1) of 69, as well as for the reverse order digits category (T1= 04; ET1= 06) (Table 1). Nevertheless, the participant presented results within the expected scoring range for the direct order digits category (T1= 20; ET1= 13). In the post-intervention assessment (T2), the results were found to be age appropriate. It is also possible to notice this skill's progress in all categories, pseudowords (T1= 66; T2= 69), direct order digits (T1= 20; T2= 24) and, particularly, in reverse order (T1= 04; T2= 12) (Table 1), which requires executive functioning aspects that assist in the rapid storage of the response, a differential aspect in high abilities.

As for the rapid automatized naming, it was observed that in T1, the performance was inadequate for the normality standards in all subtests. It is also possible to say that, in T2, the participant presented a lower-than-expected performance for the categories of digits (T2= 41; ET2= 35), objects (T2= 59; ET2= 50) and colors (T2= 56; ET2= 53). Only the category of letters presented results within the expected range (T2= 29; ET2= 32). On the other hand, the improvement in naming speed is visible for the subtests of letters (T1= 37; T2= 29), objects (T1= 62; T2= 59) and colors (T1= 60; T2= 56), except for digits (T1= 37; T2= 41) (Table 1). Regarding the stimuli's naming time decrease, it is possible to say that the child becomes more effective in accessing the mental lexicon at the level of phonological and visual representation, which is also not usual in isolated dyslexia.

On the subject of reading, the participant presented alphabetic level in T1 and, in T2, orthographic level. In the first assessment, it was observed that the difficulty was mainly with visually similar and phonologically neighbors. Furthermore, the student used sub-vocal support to decode and presented an average reading of 20 words per minute, which indicates extremely slow decoding, much lower than expected for her educational level. In the reassessment, she obtained an average of 94.4 words per minute in oral reading, considered adequate for her school year. The participant demonstrated presence of prosody, rhythm, global reading, commitment and adequate comprehension. Qualitatively, it may be noted that the child, even with adequate performance, read at a low vocal intensity, still demonstrating insecurity to perform the task.

**Figure 1:**
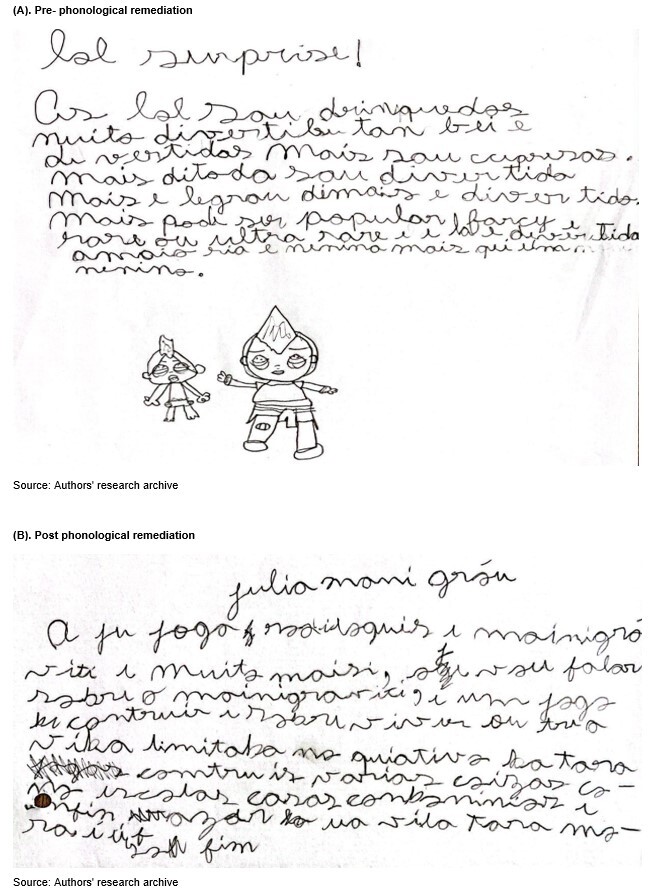
Child's writing sample

In writing, it can be observed that in T1 the child had poor pencil grip, imprecise writing, with letter replacements, omissions, hyper and hypossegmentations, word repetition and low use of cohesive elements (Figure 1A). In this period, the participant's writing was transitioning from the syllabic-alphabetic phase to the alphabetic phase. In T2, no significant change was observed, since her writing remained inaccurate, with little content intelligibility, with the replacement of visually similar letters (such as “d” and “b”) and lack of punctuation. According to the sample collected, she was in the alphabetic phase of writing, although difficulties no longer expected for her age still persisted (Figure 1B). Nevertheless, it is noted that the participant used a greater repertoire in the vocabulary for the visual input lexicon.

After analyzing the results in their entirety, it can be observed that written language skills improved during the interval between assessments, despite the permanence of characteristics associated with dyslexia, as the participant still demonstrated a lower than expected performance in lexical access and in writing - with the presence of persistent replacements between auditory and visually similar phonemes, omission of letters and hypersegmentation .

## DISCUSSION

The present case report was elaborated to demonstrate how an individual with developmental dyslexia, or any other neurodevelopmental disorder can present atypical performance, according to the variables that they are exposed to.

In other investigations, when comparing the performance of children with dyslexia only, with HA/G only and with twice exceptional children with dyslexia, it was demonstrated that the latter group showed a better overall reading performance than dyslexic children, and a lower performance than children with HA/G only^(10)^. Therefore, the performance of children with 2E and dyslexia may not be as deficient as that of children who have only a learning disability.

The use of phonological remediation programs tends to indicate a better evolution in phonological awareness and a slower progress on the reading level regarding children with dyslexia^(7,10,19)^. Thus, the reading level persistence would be expected after the proper number of interventional appointments. In this case, the child presented a change in reading level. Hence, it is possible that HA/G influenced the results positively.

As the participant had been exposed to more than one language since birth, it is equally possible that the bilingualism factor, in addition to the HA/G, has influenced her language development^(20)^. However, it does not justify the persistence of lower-than-expected performance in phonological processing and writing skills. Such abilities indicated characteristics consistent with the diagnostic criteria of dyslexia even after intervention exposure^(7)^.

Considering that phonological working memory and phonological awareness are essential factors for adequate reading performance, with the improvement of these skills there was an evolution from the alphabetic to the orthographic level, demonstrating the effectiveness of the intervention. It could be said that the child's positive response was also due to the particularity that verbal skills are better developed in twice exceptional children with dyslexia than non-verbal skills, along with better verbal comprehension, reasoning and abstract thinking^(21,22)^. Consequently, the process of building metalanguage can be facilitated when working on phonological remediation programs.

Regarding the access to the mental lexicon, there may have been a performance variation with the application of different instruments, making the comparison of pre- and post-intervention results quantitatively different. Nevertheless, it was qualitatively observed that a lexical access in a shorter time may have contributed to better decoding, since it is a predictive reading skill, even if still impaired.

Writing showed no significant improvement, since it was not the focus of the intervention. In this sense, it would be necessary to implement further intervention strategies focused on this aspect so that the quality of the child's writing did not remain below expected for their age and school year. That being said, the written language difficulty observed before and after the intervention can also be expected in individuals with twice-exceptionality (2E) associated with dyslexia. As other studies have observed, superior verbal reasoning does not exclude the impairments associated with dyslexia in writing^(23)^, which confirms another characteristic of this condition due to the alteration persistence.

Therefore, in view of the results presented herein, understanding this performance profile is fundamental. There is a possibility that the strength points of a 2E child with dyslexia may favor the development of these skills, *a posteriori*, with adequate instruction, as observed with the positive response of the present report's participant to phonological remediation.

Although the scientific community recognizes the presence of 2E students, there are still major challenges in identifying these children. A greater characterization and understanding of this condition's different profiles, as well as further instruction for the professionals who work directly with these students is necessary to identify and intervene effectively. Furthermore, it is important to consider the diversity of performance and, mainly, to work with the potentialities found in each case as a force to overcome difficulties during the learning process.

## FINAL COMMENTS

According to the findings herein, it was possible to observe that after phonological remediation there was an evolution in the skills of phonological awareness, lexical access, phonological working memory, reading level and speed. For a child diagnosed with dyslexia only, the difficulties were expected to be persistent after the time of exposure to the phonological remediation. Although the participant still demonstrated characteristics of her diagnosis after the intervention, such as underperformance in accessing the mental and written lexicon, she was able to advance in several skills, especially in reading. This result indicates that individuals with HA/G associated with dyslexia can present greater speed and engagement in interventional strategies to overcome language difficulties when compared to individuals with dyslexia alone. Additionally, it was possible to investigate these characteristics in bilingual children, bearing in mind the growth of this population in Brazil.

This way, future studies on the different types of 2E may contribute to a better understanding of this condition during the learning development, along with the specialized interventions, such as phonological remediation.
