# Supplementary material for: Phonological remediation effects on a child with giftedness and developmental dyslexia
Source: Codas. 2024 Aug 5;36(3):e20230068. doi: 10.1590/2317-1782/20242023068en (PMC11340868; doi:10.1590/2317-1782/20242023068en)
Supplement: Translation

# Translation: Efeito da remediação fonológica em criança com altas habilidades/superdotação e dislexia do desenvolvimento

**DOI:** 10.1590/2317-1782/20242023068pt

**Authors:** Alba Miranda Beserra Gurgel Sena, Bárbara Louise Costa Messias, Roberta Louise Mariano Bezerra, Anna Irenne de Lima Azevedo, Hellen França Alcantara, Cíntia Alves Salgado Azoni

**Affiliations:** 1 Programa Associado de Pós-graduação em Fonoaudiologia – PPGFon, Universidade do Rio Grande do Norte – UFRN - Natal (RN), Brasil.; 2 Programa de Pós-graduação em Psicologia, Universidade Federal do Rio Grande do Norte – UFRN - Natal (RN), Brasil.; 3 Departamento de Fonoaudiologia, Universidade Federal do Rio Grande do Norte – UFRN - Natal (RN), Brasil.

**Keywords:** Terapia da Linguagem, Relatos de Casos, Leitura, Dificuldade de Desenvolvimento de Leitura, Escrita Manual

## Abstract

A dupla-excepcionalidade é caracterizada pela presença de alto desempenho concomitante a deficiências ou condições incompatíveis, como é o caso de altas habilidades associadas a transtornos do neurodesenvolvimento. Esse estudo é um relato de caso clínico referente ao processo avaliativo e interventivo de uma criança de 9 anos com a combinação paradoxal de altas habilidades associadas à dislexia. O objetivo foi comparar o desempenho nas tarefas de processamento fonológico, leitura e escrita pré e pós remediação fonológica. Na primeira avaliação, a criança apresentou nível alfabético na leitura, fase de transição entre os níveis silábico-alfabético e alfabético na escrita e desempenho abaixo do esperado nas habilidades do processamento fonológico. Após a intervenção, houve melhora em habilidades do processamento fonológico, consolidação da escrita alfabética e do nível ortográfico de leitura. Em geral, crianças com dislexia isolada apresentam dificuldades persistentes em várias habilidades após intervenção. A evolução demonstrada após a remediação fonológica, principalmente no nível de leitura, mostra características diferentes do esperado. Assim, pode-se concluir que a dupla-excepcionalidade pode ter favorecido a superação de algumas de suas dificuldades de forma mais exitosa. Estudos sobre estas condições combinadas podem contribuir para a melhor compreensão deste quadro durante o desenvolvimento da aprendizagem e para a formulação de intervenções especializadas.

## INTRODUÇÃO

A dupla-excepcionalidade (DE) pode ser caracterizada pelo alto desempenho em uma ou mais áreas, concomitantes com uma desordem psiquiátrica, educacional, sensorial ou física. Caracteriza-se como uma construção mediada entre o potencial e a fraqueza que, de forma coexistente, produz algo qualitativamente diverso^(1)^. Nesse sentido, crianças podem apresentar conjuntamente altas habilidades/superdotação (AH/S) e transtornos do neurodesenvolvimento, como: Transtorno de déficit de atenção e hiperatividade (TDAH), Transtorno do espectro autista (TEA), Transtornos de aprendizagem (TA), dentre outros^(2)^.

A última década foi marcada pela dificuldade em reconhecer crianças e adolescentes com dupla-excepcionalidade, especialmente devido aos mitos e ideais mal concebidos na sociedade que envolvem as altas habilidades/superdotação (AH/S)^(3)^.

Historicamente, as AH/S podem apresentar diferentes modelos teóricos utilizados para sua compreensão. O modelo dos três anéis de Renzulli é um dos mais significativos na contribuição do estudo das AH/S e será utilizado neste relato de caso para compreender as AH/S. Nessa concepção teórica, as AH/S são compreendidas a partir de 3 eixos: criatividade, inteligência (capacidade acima da média) e comprometimento com a tarefa^(4,5)^.

No presente relato, será evidenciada a combinação paradoxal entre as AH/S e um quadro de transtorno específico de aprendizagem com prejuízo na leitura, a dislexia.

A dislexia é caracterizada dentro do grupo de Transtornos Específicos de Aprendizagem. No caso do transtorno específico de aprendizagem com prejuízo na leitura, ou dislexia, a criança pode apresentar prejuízos persistentes na precisão da leitura de palavras, fluência e compreensão leitora, velocidade da leitura, escrita e dificuldades na ortografia^(6)^. Como tal, essa condição apresenta habilidades acadêmicas demasiado abaixo da média para a idade e escolaridade^(7)^.

Assim, para compreender com mais precisão o desempenho e a dificuldade da criança nas referidas habilidades de leitura, pode-se delinear o nível de leitura que o aluno se encontra a partir da teoria de Frith, respectivamente dividido nos níveis: logográfico, alfabético e ortográfico^(8)^.

Crianças que apresentam dupla-excepcionalidade podem evidenciar desempenho acadêmico, cognitivo e social qualitativamente diferente de crianças que apresentam quadro clínico apenas de AH/S ou dislexia separadamente. Sendo o desempenho diverso e diferente para cada caso clínico, ainda assim, quando essas crianças são avaliadas, a literatura aponta para talentos específicos como: alto nível de criatividade, imaginação incomum e habilidades intelectuais de nível superior. Entretanto, ao mesmo tempo, podem ser observados déficits em leitura e escrita^(2,9)^.

Investigações têm observado que, frequentemente, num quadro de DE com dislexia, as AH/S podem mascarar o baixo desempenho em habilidades acadêmicas. Assim, as potencialidades podem auxiliar a superar as dificuldades apresentadas^(2,10)^. Deste modo, por se tratar de uma área incipiente, com ausência de uniformidade em avaliação e intervenção, esses fatores contribuem para o desafio de identificação e estratégias especializadas, pois é marcada por uma forte heterogeneidade de desempenho. É essencial compreender quais estratégias podem ser mais eficazes como prática interventiva para as competências dessas crianças para que possam superar suas maiores fragilidades e reconhecer possíveis pontos de força.

Dessa forma, o objetivo do presente estudo é comparar o desempenho de uma criança com altas habilidades/superdotação (AH/S) associado a dislexia nas tarefas de processamento fonológico, leitura e escrita pré e pós intervenção baseada em um programa de remediação fonológica. Com isso, será possível gerar novas reflexões sobre a temática e auxiliar na identificação de possíveis desafios encontrados para indivíduos duplamente excepcionais.

## APRESENTAÇÃO DO CASO CLÍNICO

Trata-se de um estudo de caso, aprovado pelo Comitê de Ética em Pesquisa (CEP) sob número 1.012.635. A autorização prévia dos familiares e da participante foi solicitada a partir da assinatura do Termo de Consentimento Livre e Esclarecido (TCLE) e do Termo de Assentimento Livre e Esclarecido (TALE).

A participante desse estudo é uma estudante do 3º ano do Ensino Fundamental do sexo feminino. Na primeira avaliação, em 2018, a criança estava com 8 anos e 2 meses, enquanto na segunda avaliação, em 2019, se encontrava com 9 anos e 6 meses. O intervalo entre as avaliações ocorreu por tratar-se de um serviço público. Assim, o laboratório se ausentou das atividades durante as férias. Além disso, é importante considerar que os atendimentos foram realizados apenas uma vez por semana e, durante esse período, a participante apresentou faltas, o que também prolongou o processo. Quanto ao seu histórico, nasceu a termo e apresentou desenvolvimento neuropsicomotor e linguísticos adequados. A criança nasceu e morou em um país de língua francesa até os 2 anos de idade, mas possuía exposição a outro idioma em casa, visto que seus pais são falantes do português brasileiro. Contudo, suas primeiras palavras foram no idioma francês. Ao retornar ao Brasil, passou por duas escolas privadas. Na primeira escola, não conseguia se comunicar, pois se expressava apenas em francês. Após essa experiência, aos 3 anos, passou a estudar em uma escola francesa, ainda no Brasil. Com o decorrer dos anos, apresentou dificuldade na aquisição da leitura e escrita; por esse motivo, repetiu o 1º ano do Ensino Fundamental, a pedido da mãe. Aos 6 anos, passou a estudar em uma escola bilíngue português-inglês. Aos 8 anos de idade, realizou avaliação por equipe interdisciplinar nas áreas de fonoaudiologia e neuropsicologia, constatando o diagnóstico de dislexia do desenvolvimento (DD) e altas habilidades/superdotação (AH/S). Logo após, foi encaminhada para a avaliação de leitura e escrita no laboratório de Linguagem Escrita, Interdisciplinaridade e Aprendizagem - LEIA/UFRN.

Fases do estudo: quatro sessões de avaliação para cada momento - pré e pós-intervenção (T1 e T2, respectivamente)- e 20 sessões de remediação fonológica, uma vez por semana durante 60 minutos. A intervenção ocorreu no segundo semestre de 2018, onde os pais se mostraram pouco engajados devido à demanda de trabalho.

As avaliações foram realizadas individualmente com duração de uma hora cada. Nelas, foram realizadas tarefas para avaliar o desempenho em processamento fonológico - memória de trabalho fonológica, consciência fonológica e acesso ao léxico mental -, leitura e escrita.

Para a avaliação da criança, foram utilizados os seguintes protocolos:

- **Consciência fonológica:** para avaliar esta habilidade, foi utilizado o Teste de Consciência Fonológica Instrumento de Avaliação Sequencial - CONFIAS^(11)^. Esse protocolo propõe tarefas de síntese, segmentação, rima, aliteração, identificação de sílaba inicial e final, exclusão e transposição. Primeiramente, analisa-se a consciência silábica, formada por nove itens, logo após, analisa-se a consciência fonêmica, formada por sete itens. Cada acerto equivale a um ponto, sendo 40 o total da consciência silábica e 30 o da fonêmica, totalizando 70 pontos. Seus resultados devem ser comparados com as hipóteses de escrita esperadas baseadas em Ferreiro e Teberosky^(12)^. Dessa maneira, utilizou-se os seguintes valores de normalidade: para hipótese de escrita silábica-alfabética, 27, 12 e 39 para o nível silábico, fonêmico e pontuação total, respectivamente; para hipótese de escrita alfabética, 31, 15 e 46.
- **Memória de trabalho fonológica:** foi aplicada a Prova de Memória de Trabalho Fonológica^(13)^. Na aplicação deste protocolo, o avaliador deve começar pela prova de não-palavras, que é composta por 40 palavras inventadas. Assim, deve falar cada palavra que compõe a lista, pedindo para a criança repeti-las de forma imediata. A criança terá duas tentativas para repetir adequadamente. Na primeira tentativa, cada acerto equivale a dois pontos, caso acerte na segunda, recebe um ponto e, por fim, atribui-se zero quando não conseguir em nenhuma das tentativas. Após isso, o avaliador deve seguir para a prova de dígitos de ordem direta e inversa, que tem a forma de pontuação equivalente à das pseudopalavras. Conforme a idade da participante no momento das avaliações, utilizou-se os valores de normalidade de 69, 13 e 6 nas pseudopalavras, dígitos diretos e inversos, respectivamente.
- **Acesso ao léxico mental:** utilizou-se o Teste de Nomeação Automática Rápida (RAN)^(14)^ na avaliação e o Teste de Nomeação Automática (TENA)^(15)^ na reavaliação. Ambas as provas têm como objetivo estimar a capacidade do indivíduo em nomear uma sequência de estímulos, ou seja, irá medir a velocidade que a criança exibe em verbalizar um estímulo visual de forma rápida. Houve uso de dois protocolos, visto que na época da primeira avaliação o TENA ainda não havia sido publicado. Além disso, o TENA é um protocolo atual e mais completo para a verificação da normalidade, pois possibilita análise de acordo com idade e meses. Os dois testes utilizados possuem aplicação semelhante e são divididos em quatro pranchas, onde a criança deve nomear cores, objetos, letras e dígitos. A nomeação deve ser feita com o mesmo movimento que se faz para a leitura - da esquerda para a direita e de cima para baixo. Para T1, a qual utilizou-se o RAN, os valores de normalidade correspondem a crianças com idade entre 8 anos e 8 anos e 11 meses, em virtude da idade da participante nesse período, assim, deve apresentar pontuação de 28, 29, 52 e 46 segundos para os subtestes de dígitos, letras, objetos e cores, respectivamente. Para T2, utilizou-se os valores de normalidade da idade de 9 anos e 6 meses do protocolo (TENA), com pontuação esperada de 35, 32, 50, 53 segundos para os subtestes de dígitos, letras, objetos e cores, respectivamente.
- **Leitura:** primeiramente, foi utilizado o protocolo de Avaliação da Leitura de Palavras/pseudopalavras Isoladas – LPI^(16)^, no qual é solicitado que a criança leia em voz alta palavras e pseudopalavras isoladas, que serão pontuadas. São dispostas 19 palavras regulares, 20 irregulares e 20 pseudopalavras em arial preta, tamanho 24 e fundo branco. A criança poderá obter um total de 59 pontos, visto que cada leitura correta equivale a um ponto. Após esse momento, foi utilizado o Protocolo de Avaliação da Compreensão Leitora de Textos Expositivos^(17)^. Este instrumento tem como objetivo avaliar a compreensão leitora por meio de perguntas direcionadas sobre textos compatíveis ao ano escolar do sujeito. Nele são avaliados e cronometrados os padrões de leitura silenciosa e oral. Com isso, pode-se verificar qual o nível de leitura que se encontra. Além disso, é realizada a média do número de palavras lidas por minuto, possibilitando a verificação e comparação da velocidade de leitura.
- **Escrita:** para avaliar a escrita, foi solicitado que a criança produzisse um texto com tema de seu interesse. Após finalizar a história, o profissional pediu à criança para que lesse em voz alta o que foi escrito. Ademais, foi solicitado que a criança escrevesse em folha avulsa as palavras-alvo do LPI^(16)^, de modo a realizar um ditado de palavras e pseudopalavras. Com isso, foi realizada uma investigação da escrita de forma qualitativa, baseada na análise ortográfica de Zorzi e Ciasca^(18)^.

A remediação realizada foi baseada em um programa utilizado para crianças com dislexia^(19)^ e contou com atividades que visavam a melhora das habilidades fonológicas, tais como: identificação de grafemas e fonemas, pares de fonemas, pares de sílabas, pares de palavras, adição e subtração de fonemas, manipulação silábica e fonêmica, rima, aliteração, acesso ao léxico mental, memória de trabalho visual, memória de trabalho auditiva e treino de leitura. Em todas as sessões eram exploradas estas atividades de forma lúdica, direcionada principalmente para os aspectos metalinguísticos da consciência fonológica. No treino de leitura, a criança era exposta a livros infantis da coleção Mico Maneco. Essa coleção possui variadas histórias que aumentam o nível de complexidade das palavras, assim é possível seguir o avanço da criança. As atividades realizadas e o registro de evolução da criança eram descritos em seu prontuário no final de cada sessão.

Analisando os resultados encontrados, no que diz respeito ao desempenho em consciência fonológica, em ambas avaliações a criança apresentou desempenho consonante com as hipóteses de escrita apresentadas em cada período. Na primeira avaliação, recebeu hipótese de escrita silábico-alfabética e na segunda, alfabética, demonstrando avanço. O escore de desempenho avançou em ambas as categorias da habilidade, silábica (T1= 35; T2 = 37) e fonêmica (T1= 14; T2= 20) (Tabela 1). Destaca-se o avanço de 4 acertos no nível fonêmico, que pode ser explicado devido a remediação fonológica ter sido realizada com foco no nível fonêmico.

**Tabela 1:** Desempenho em consciência fonológica, memória de trabalho e acesso ao léxico mental

Habilidade	Variável	T1	ET1	T2	ET2
**Consciência fonológica**	Consciência silábica	35	27	37	31
	Consciência fonêmica	14	12	20	15
	Total	49	39	57	46
**Memória de trabalho**	Pseudopalavras	66	69	69	69
	Dígitos ordem direta	20	13	24	13
	Dígitos ordem inversa	4	6	12	6
Acesso ao léxico mental*	Dígitos	37	28	41	35
	Letras	37	29	29	32
	Objetos	62	52	59	50
	Cores	60	46	56	53

Legenda: T1 = desempenho na avaliação; ET1 = desempenho esperado na avaliação; T2 = desempenho na reavaliação; ET2 = desempenho esperado na reavaliação; *medida em tempo por segundos

Os resultados da memória de trabalho fonológica no momento anterior à remediação fonológica exprimem desempenho abaixo do esperado para categoria de pseudopalavras, apresentando 66 pontos em T1, com desempenho esperado para T1 (ET1) de 69, e para a categoria de dígitos na ordem inversa (T1= 04; ET1= 06) (Tabela 1). Apesar disso, apresentou resultados dentro do esperado para a categoria de dígitos na ordem direta (T1= 20; ET1= 13). Na avaliação pós-intervenção (T2), os resultados passam a se encontrar adequados para a idade. Também é possível perceber avanços nessa habilidade em todas as categorias, pseudopalavras (T1= 66; T2= 69), dígitos ordem direta (T1= 20; T2= 24) e, especialmente, para ordem inversa (T1= 04; T2= 12) (Tabela 1), que requer aspectos das funções executivas que auxiliam no armazenamento rápido da resposta, aspecto diferencial nas altas habilidades.

Quanto à nomeação automática rápida, nota-se que em T1, o desempenho encontra-se inadequado para os padrões de normalidade em todos os subtestes. Também é possível dizer que, em T2, apresentou desempenho abaixo do esperado para as categorias de dígitos (T2= 41; ET2= 35), objetos (T2= 59; ET2= 50) e cores (T2= 56; ET2= 53). Apenas a categoria de letras apresentou resultados dentro do esperado (T2= 29; ET2= 32). Por outro lado, o avanço na velocidade de nomeação é visível para os subtestes de letras (T1= 37; T2= 29), objetos (T1= 62; T2= 59) e cores (T1= 60; T2= 56), com exceção para o de dígitos (T1= 37; T2= 41) (Tabela 1). Com a diminuição do tempo de nomeação dos estímulos, é possível dizer que a criança torna-se mais eficaz para acessar o léxico mental no nível da representação fonológica e visual, o que também não é usual na dislexia isolada.

No que diz respeito à leitura, em T1 apresentou nível alfabético e, em T2, nível ortográfico. Na primeira realização do teste, notou-se que existia dificuldade principalmente com letras visualmente parecidas e fonologicamente próximas. Ademais, a estudante utilizou-se de apoio sub-vocal para decodificar e apresentou média de leitura de 20 palavras por minuto, o que demonstra decodificação extremamente lentificada e muito aquém do esperado para sua escolaridade. Já na reavaliação, obteve média de 94,4 palavras por minuto na leitura oral, considerada adequada para sua escolaridade. Apresentou presença de prosódia, ritmo, leitura global, interesse e compreensão adequada. Qualitativamente, observa-se que a criança, mesmo com desempenho adequado, leu em intensidade de fala baixa, ainda demonstrando insegurança para realizar a tarefa.

Na escrita, pode-se observar que em T1 a criança possuía preensão inadequada do lápis, escrita imprecisa, com trocas de letras, omissões, hiper e hipossegmentações, repetição de palavras e baixo uso de elementos coesivos (Figura 1A). Nesse período, se mostrava com escrita na transição da fase silábica-alfabética para a fase alfabética. Em T2 não foi observada mudança significativa, visto que sua escrita continuou imprecisa, com pouca inteligibilidade do conteúdo, trocas de letras visualmente similares (como “d” e “b”) e carência de pontuação. De acordo com a amostra coletada, se mostrou na fase alfabética de escrita, embora dificuldades não mais esperadas para sua idade persistiram (Figura 1B). Apesar disso, nota-se que utilizou maior repertório no uso de vocabulário para o léxico de input visual.

**Figura 1:** Amostra de escrita da criança

Após a análise dos resultados em sua totalidade, pode-se observar que as habilidades da linguagem escrita avançaram durante o intervalo entre as avaliações, apesar da permanência de características consonantes com a dislexia, pois ainda apresenta desempenho aquém do esperado no acesso ao léxico mental e na escrita - com a presença de trocas entre fonemas auditiva e visualmente semelhantes de forma persistente, omissão de letras e hipersegmentação.

## DISCUSSÃO

O relato de caso foi realizado com o intuito de demonstrar como um indivíduo com dislexia do desenvolvimento ou qualquer outro transtorno do neurodesenvolvimento pode apresentar desempenho atípico, de acordo com as variáveis que se é exposto.

Em outras investigações, ao comparar o desempenho de crianças apenas com dislexia, somente com AH/S e crianças duplamente excepcionais com dislexia, demonstraram que este último grupo apresentou melhor desempenho geral na leitura em comparação com crianças disléxicas, e desempenho inferior a crianças com apenas AH/S^(10)^. Desta forma, o desempenho de crianças com DE e dislexia pode não ser tão deficitário quanto o de crianças que apresentam unicamente transtorno de aprendizagem.

O uso de programas de remediação fonológica tende a mostrar melhor evolução em consciência fonológica e lentidão no avanço do nível de leitura para crianças com dislexia^(7,10,19)^. Desta forma, a persistência do nível de leitura seria esperado após o número de sessões interventivas oferecido. Neste caso, a criança apresentou mudança de nível de leitura. Portanto, possivelmente as AH/S influenciaram positivamente os resultados encontrados.

Por se tratar de uma criança que foi exposta a mais de uma língua desde seu nascimento, é possível que o fator bilinguismo, além das AH/S, tenha influenciado o seu desenvolvimento de linguagem^(20)^. Entretanto, não justifica a persistência do desempenho abaixo do esperado em habilidades do processamento fonológico e escrita. Tais habilidades demonstraram características consonantes com critérios diagnósticos de dislexia mesmo após exposição à intervenção^(7)^.

Considerando que a memória de trabalho fonológica e consciência fonológica são fatores essenciais para o adequado desempenho da leitura, com a evolução dessas competências, houve a progressão do nível alfabético para o ortográfico, demonstrando a eficácia da intervenção. Possivelmente, a positiva resposta da criança também deve-se ao aspecto de que as habilidades verbais são melhor desenvolvidas em crianças duplamente excepcionais com dislexia do que as habilidades não-verbais, além da melhor compreensão verbal, raciocínio e pensamento abstrato^(21,22)^. Assim, o processo de construção da sua metalinguagem pode ser facilitado quando trabalhado em programas de remediação fonológica.

Para o acesso ao léxico mental, pode ter acontecido uma variação do desempenho com a aplicação de instrumentos distintos, tornando a comparação dos resultados pré e pós intervenção quantitativamente distinta. Porém, qualitativamente, observa-se que o acesso ao léxico mental em tempo menor pode ter colaborado para uma melhor decodificação, visto que é uma habilidade preditora de leitura, mesmo que ainda prejudicado.

A escrita não apresentou melhora significativa, posto que não foi o foco da intervenção. Nesse sentido, seria necessário realizar estratégias interventivas focadas nesse aspecto para que a qualidade da escrita da criança não permanecesse abaixo do nível esperado para sua idade e ano escolar. No entanto, a dificuldade na linguagem escrita observada antes e após a intervenção também pode ser esperada na dupla excepcionalidade (DE) associada à dislexia. Como outros estudos observaram, o raciocínio verbal superior não exclui prejuízos associados à dislexia na escrita^(23)^, o que confirma mais uma característica do quadro pela persistência de alteração.

Assim, compreender esse perfil de desempenho torna-se fundamental diante dos resultados apresentados. Há a possibilidade de que os pontos de força de uma criança DE com dislexia possam favorecer o desenvolvimento dessas habilidades, a *posteriori,* com a instrução adequada, como observada pela criança no presente relato ao responder de maneira positiva à remediação fonológica.

Embora a comunidade científica reconheça a presença de alunos com DE, ainda existem grandes desafios na identificação dessas crianças. É necessária maior caracterização e compreensão dos diferentes perfis nesta condição e instrução dos profissionais que trabalham diretamente com esses alunos para identificar e intervir de maneira efetiva. Outrossim, é importante considerar a diversidade de desempenho e, principalmente, trabalhar com as potencialidades encontradas em cada caso como força de superação das dificuldades durante o processo de aprendizagem.

## COMENTÁRIOS FINAIS

Diante dos achados, foi possível observar que houve evolução após a remediação fonológica nas habilidades de consciência fonológica, acesso ao léxico mental, memória de trabalho fonológica, nível e velocidade de leitura. Para uma criança com diagnóstico de dislexia isolada, era esperado que as dificuldades se mostrassem persistentes após o tempo de exposição à remediação fonológica. Apesar de ainda demonstrar características de seu diagnóstico após a intervenção, como o desempenho aquém do esperado no acesso ao léxico mental e escrita, foi capaz de avançar em várias habilidades, principalmente no âmbito da leitura. Tal resultado demonstra que indivíduos com AH/S associadas a dislexia podem apresentar maior rapidez e engajamento em estratégias interventivas para a superação de dificuldades de linguagem quando comparadas à dislexia de maneira isolada. Além disso, investigar estas características em crianças bilíngues, considerando o crescimento desta população no Brasil.

Dessa forma, estudos futuros acerca dos diferentes tipos de DE podem contribuir para a melhor compreensão deste quadro durante o desenvolvimento da aprendizagem e intervenções especializadas, como a remediação fonológica.
